# REDUCING BENEVOLENT AGEISM AMONG UNDERGRADUATE HEALTHCARE MAJOR STUDENTS IN A GERONTOLOGY CLASS

**DOI:** 10.1093/geroni/igad104.3771

**Published:** 2023-12-21

**Authors:** Jiawei Cao, Jialin Cui

**Affiliations:** Nanjing University of Chinese Medicine, Nanjing, Jiangsu, China (People’s Republic); Nanjing University of Chinese Medicine, Nanjing, Jiangsu, China (People’s Republic)

## Abstract

While gerontologists have devoted continuing efforts to combating ageism through education and interventions, benevolent ageism remains to be a more subtle and covert concept to understand and recognize. This study aims to examine the effectiveness of an innovative pedagogy to reduce benevolent ageism among undergraduates majoring in healthcare with an aging focus. Two classes (36 students in the intervention group and 43 in the control group) that were both enrolled in an entry-level gerontology class were recruited. The intervention consisted of a semester-long synchronized lecture with pre-lecture quizzes, videos, examples, and in-class group projects regarding benevolent ageism, while the control group received a regular gerontology class with no additional emphasis on ageism. At the beginning and the end of the semester, all participants completed a questionnaire that was built upon the translated Ambivalent Ageism Scale (AAS) benevolent subscale. Mixed-design ANOVA was conducted to examine the effects of the pedagogy (intervention vs. control) and time (pre-test and post-test) on students’ benevolent score. The score for both groups did not differ at pre-test, and the results showed a significant interaction of group by time (F (1, 77) =13.24, p < .001). The benevolent subscale score of the intervention group decreased significantly over time, while the control group only showed a slight but insignificant decrease in the score. Findings from our study suggested that this innovative pedagogy may be useful in reducing benevolent ageism among healthcare major undergraduates. However, further randomized studies are needed to confirm our findings.

